# Medical Education and State License Examinations

**Published:** 1897-06

**Authors:** William Warren Potter

**Affiliations:** Buffalo, N. Y.; Examiner in obstetrics, New York State Medical Examining and Licensing Board


### MEDICAL EDUCATION AND STATE LICENSE EXAMINATIONS.

Conducted by WILLIAM WARREN POTTER, M. D., Buffalo, N. Y.,
Examiner in obstetrics, New York State Medical Examining and Licensing Board.

THOMAS McDAVITT, M. D., of St. Paul, read a paper before the Minnesota State Medical Society, June 17, 1896 (*Northwestern Lancet*), in which he gave a retrospective review of the medical legislation in the United States. He said less than twenty years ago practically any one could practise medicine anywhere in the United States. Owing to the efforts of the Illinois State Board of Health, which were only the crystallised ideas of its lamented secretary, John H. Rauch, the idea became a working force that a change was necessary in the manner of admitting M. D.'s to practice. Practice had demonstrated that the average medical college was only a diploma mill. A great majority were run on purely a commercial basis. Many were stock concerns, the stock owned by the different professors, and the more matriculants the more dollars. The more matriculants permitted a diploma this year the more matriculants next year. The announcements of most colleges, theoretically, asked preliminaries altogether too low, and asked no questions at all when the party matriculated. In days not far back two winter terms of five months each brought the legal qualifications to deplete the population, and we now look back with shame to the time when an ignorant individual, never having looked into a medical book previously, could take one winter term of five months and immediately go to a spring term school and come out in the early summer a full-fledged, duly-authorised M. D., and could force his ignorant and unworthy self on any community of this country.

The earliest effort at increasing the time of study was due to the efforts of that "grand old man," N. S. Davis, at the organisation of the Chicago Medical College in 1857 or 1858. The faculty advised three terms, but permitted two. Each year saw an increasing number taking three terms. Soon Harvard came into line, urging three terms, and very soon afterward demanding three terms. The low grade schools were, however, manufacturing doctors by the score and the suffering public was forced to submit. The efforts of the Illinois board to attempt to a certain extent to throw safeguards around the public, in the way of looking into the genuineness of the different diplomas held by so-called M. D.'s, were met with hostility not only by the quacks and ignoramuses, but many medical schools fought the idea that they should be under any supervision whatever and their own sweet will should be the only determining factor.

Previous to this time ideas on this question were at such a pass that a number of so-called schools absolutely sold diplomas for so much cash without the possessor ever having seen the inside of a college. These fake diploma shops were finally shut up, but much sympathy was expended on them. Many of them were closed by the admirable and far-reaching work of the Illinois board, and, as said before, it may be regarded as pioneers in medical legislation.

Virginia and North Carolina introduced legislation quite early. Minnesota followed with her law of 1883, which accepted diplomas. It was soon evident that legislation was necessary before many of the so-called first-class colleges would ever increase their curricula. Our state had suffered from the effect of poor M. D.'s, from poorer medical colleges, and our best men recognised that a change must be made at the fountain-head—the medical colleges—although it might be unfair to a few of their graduates. Several of our best men, by the force of their arguments, got the law of 1887 passed, even against the opposition of many of the legislators and, undoubtedly, many of the people. This law made three regular terms at a medical college, of at least six months each, necessary before the candidate was even eligible for examination. This was executed without fear or favor and, while it prevented many from taking the examination, in the end it accomplished what was intended—namely, raised the standard to three years. The feeling exhibited by many colleges was intense and two of the largest pooled their issues, retained counsel to fight the law, but said counsel soon advised dropping the fight. Almost all points of our law have been decided by the supreme court and declared constitutional. At our last legislature an amendment was passed increasing the terms necessary before a party was eligible for examination to four terms after 1899. Four terms are also necessary in the Medical College Association before a college can become a member.

The great point now is to force a certain amount of preliminary education before a person is eligible for matriculation in a college, and it appears that legislation will be necessary, and its execution placed in the hands of the different boards, before many colleges will adopt it. The lukewarmness with which many colleges look upon any efforts to increase the standard, or even maintain it, is one of the crying evils of the time. There is no denying the fact that the most potent element of evil in medical education at present is the unnecessary number of the medical colleges. What right many of them have of existing is an enigma. We all know of many examples of so-called colleges without apparatus, or facilities for teaching, generally located in small-sized towns, without adequate clinical advantages. In another city of 400,000 or 500,000 twelve or fifteen medical colleges can be pointed out. In our whole country, 140. The great medical need of the country is the revoking of numerous charters, the increasing of the standard of those which remain, and the elevating of our profession to that of a learned profession. The effort to force the colleges to a higher preliminary standard, as well as a four-term course (in accordance with our Minnesota law), is indicated in the following resolutions by the Illinois state board :

RESOLUTIONS OF ILLINOIS STATE BOARD OF HEALTH.

Conditions of admission to lecture courses :

*First.*—Credible certificate of good moral character signed by two physicians of good standing in the state from which the applicant comes.

*Second.*—A diploma or certificate of graduation from a high school; evidence of having passed the matriculation examination to a recognised literary or scientific college, or a certificate of successful examination by the faculty of any reputable university or college, or by the state superintendent of public instruction in the following branches: English grammar, arithmetic, elementary, physics, United States history, geography, Latin (equivalent to one year in a high school). One year is allowable in which to cure defects in knowledge of Latin, but the student must be provided with a certificate of proficiency in this branch of learning from the designated authorities before he can be accepted as a second course student.

*Resolved*, That graduates of dental surgery, pharmacy and veterinary medicine may be entitled to credit for one year's study in said recognised colleges, on complying with their entrance requirements and passing examinations in all the branches embraced in the previous years of study.

*Resolved*, That as a further condition of the recognition of any medical college as in good standing the college shall furnish the secretary of the State Board of Health or State Board of Medical Examiners, on or before January 1st of each year, a complete list of all its matriculants, together with the basis upon which each applicant matriculated, giving the name of the institution from which the degree or certificate of graduation was obtained, or the name of the state official conducting the examination, or the university or college conducting examinations, or the college previously attended, together with the date when the degree or certificate was issued. This list to be sworn to by the executive officer of the college and attested by the secretary, under the seal of the college.

*Resolved*, That students who have heretofore attended full courses of instruction in non-recognised medical colleges may be admitted to advanced standing in recognised medical colleges, complying with the entrance requirements of said recognised medical colleges, and passing other examinations in all the branches in the previous years of study.

*Resolved*, That no medical college shall be recognised as in good standing for the purposes of the Illinois Medical Practice Act that does not require of all matriculants after January 1, 1897, as a condition of graduation, four (4) full courses of lectures in four (4) separate years.

The synopsis of present states with their legislation indicates the change of less than twenty years from no legislation until there are only five or six lagging behind. The statistics are partly due to an article in the *Bulletin of American Academy of Medicine*, by Dr. Charles McIntire, Easton, Pa. Although written in December, 1895, there have been laws passed in some places which had none then.

TABLE I.

*a*. States and territories whose medical laws practically permit any one to open an office to practise medicine :

Alaska, Arizona, Kansas, Michigan, Nevada, Texas, Wisconsin and Wyoming.

*b.* States and territories which merely exercise a supervision of the diploma held by the person desiring to practise medicine :

California, Kentucky, Missouri and Nebraska.

*c.* States and territories which require an examination for license, but accept the diplomas of certain colleges as evidence of that examination :[1]

District of Columbia, Ohio, Arkansas, Colorado, Connecticut, Illinois, Indian territory, Chocktaw Nation, Florida[5], Iowa, Massachusetts, New Mexico, Oklahoma, Rhode Island, South Dakota, Tennessee, Vermont, Idaho, Indiana and New Hampshire.

*d.* States and territories that require passing an examination before a state board of examiners before issuing a license to practise medicine :[2]

1. A single board of examiners :

Alabama,[3] Indian Territory, Cherokee Nation,[4] Maine, Minnesota, Mississippi, Montana, New Jersey, North Carolina, North Dakota, Oregon, South Carolina, Utah, Virginia, Washington, West Virginia.

2. Two or three boards of examiners, one of them homeopathic and another eclectic :

Georgia, New York and Pennsylvania.

*e.* States from whom no reply has been received to letters of inquiry :

Indian Territory, Creek Nation.

---

### THE STATE BOARD OF PHARMACY.

ONE hundred and sixty-six candidates (*Northern Druggist*, May, 1897,) appeared before the board for examination on February 19th, and of this number sixty-three were passed and 103 were rejected. Of the successful candidates fifty were licensed as pharmacists and thirteen as assistant pharmacists.

---

1. It must be remembered that each state determines for itself the colleges whose diplomas will be accepted.

2. In some states the candidates for examination must possess the certificate of graduation from a medical school (if an American school the degree of M. D.); other states make the examination the sole criterion of fitness to practise.

3. Each county has its board of examiners

4. There is a board for each judicial district.

5. Florida has one board for each judicial district and a homeopathic board for the state at large.

### MEDICAL STUDENT EXAMINATIONS IN ILLINOIS.

THE Illinois State Board of Health no longer allows medical colleges to examine students in general education on matriculating. If the prospective medical student is not a graduate of a high school or some institution of equal rank, or has not passed the entrance examination to some recognised literary college or university, he must submit to an examination by the faculty of some reputable literary college or university in the common English branches and in first year Latin.

---

### TENNESSEE AND CHRISTIAN SCIENCE.

IN A BILL proposing amendments to the Tennessee medical practice act we note the following:

Section 8. Be it further enacted, That any person shall be regarded as practising medicine or surgery, within the meaning of this act, who shall use the words or letters, "Dr." "Doctor," "Professor," "M. D.," or "M. B.," in connection with his or her name, or any other title intending to imply or designate him or her as a practitioner of medicine or surgery in any of its branches, and who, in connection with such title, or titles, or without the use of such titles, shall prescribe, direct, recommend, advise, apply, give, or sell, for the use of any person or persons, any drug or medicine, or other agency or application for the treatment, cure or relief of any bodily infirmity, injury or disease; and it is further provided that the use of any one of the aforementioned titles, or the exposure of a sign, circular, advertisement or any other device or information, indicating thereby the occupation of the person or persons, shall be considered *prima facie* evidence; and it is further provided that the provisions of this act shall apply to all persons professing and attempting to cure diseases by means of the so-called systems of "faith-curism," "mind-healing," "laying-on-of-hands" and all other similar systems.

This is the first attempt to dispose of christian science fiends by legislative enactment that we have observed. We understand the amendments have been passed.

---

### IDAHO PASSES A GOOD MEDICAL LAW.

IDAHO secured a Medical Examining Board and law March 8, 1897, (*Indiana Medical Journal,*) during the expiring hours of the session, by a unique ruse. The State Medical Society purchased a diploma from the Rutland, Wis., diploma mill outfit for $35.00, which the Hon. J. J. Rogers whipped out and triumphantly waved before the house when it was contended that a diploma was enough to satisfy any reasonable requirements. When Representative

Rogers asserted he had just been made an "M. D." for so small an expenditure of time and money, the hordes of quacks opposing the bill were paralysed, and the members fell over each other in their desire to vote for the bill. Governor Steunenberg declined to veto the bill and it is a law. The board has three regulars, two eclectics and one homeopath. The license fee is $5. The board may refuse license for unprofessional, criminal, immoral or dishonorable conduct.

Unprofessional or dishonorable conduct is defined to be abortion work; employment of cappers or steerers; taking a fee for a promise to cure an incurable disease; wilful betrayal of a professional secret; advertising untruthful or improbable claims; advertising to restore the menstrual flow; conviction of criminal offense; habitual intemperance in stimulants and drugs. The penalty for violation is $50 to $300, or ten days to six months, or both. The law is far more stringent than that of Indiana.

Itinerant vendors of any drug, nostrum, ointment or other remedy, appliance or apparatus, or anyone printing, publishing or claiming publicly or privately to treat, palliate or cure disease, injury, deformity or defect by drugs, nostrum, manipulation or other expedient or apparatus, for gain, directly or indirectly, shall first procure a license, paying $50 per month. Such fund goes to the school fund. Penalty is provided for evasion of this provision. Traveling eye specialists, spectacle vendors and opticians are classed under this provision.

The present act was passed upon and approved by the joint judiciary committee of the two houses, and is no doubt constitutional. It was fought by all the quacks in the state, and even a few misguided practitioners of standing. Money was plenty to destroy it, if possible.

Major W. M. Wood, M. D., U. S. A., present president and chairman of the committee on judiciary of the Idaho State Medical Society, had prepared an unanswerable memorial from the society, which, at the appropriate time, was presented to the legislature. This had been carefully printed, and its distribution made many converts. In fact, if it had not been for the use of money, it is doubtful if there would have been any answer made to the arguments. As it was, the quack interest was on hand strong and powerful, and Dr. Sweet and Dr. Wood, who marshaled the outside forces, went into the fight of their lives, which lasted throughout the entire sixty days' session, and up till 2 A. M. the last night, and then only won as the legislature was closing.

